# Psychometric assessment of the 10-item, revised experience of close relationship (ECR-R-10) in nonclinical and clinical populations of adults and older adults in Thailand

**DOI:** 10.1038/s41598-023-41306-y

**Published:** 2023-09-11

**Authors:** Nahathai Wongpakaran, Tinakon Wongpakaran, Peerasak Lerttrakarnnon, Surin Jiraniramai, Nattha Saisavoey, Thanita Tantrarungroj, Sirina Satthapisit, Justin DeMaranville, Khin Moe Myint, Danny Wedding

**Affiliations:** 1 Psychotherapy Unit and Geriatric Psychiatry Unit, Department of Psychiatry, Faculty of Medicine, Chiang Mai University, 110 Intawaroros Road, T. Sriphum, A. Muang, Chiang Mai, 50200 Thailand; 2 Department of Family Medicine, Faculty of Medicine, Chiang Mai University, Chiang Mai, Thailand; 3 Department of Psychiatry, Faculty of Medicine, Siriraj Hospital, Mahidol University, Bangkok, Thailand; 4 Department of Psychiatry, Faculty of Medicine, Ramathibodi Hospital, Mahidol University, Bangkok, Thailand; 5 KhonKaen Hospital, Khon Kaen, Thailand; 6 Graduate School, Chiang Mai University, Chiang Mai, 50200 Thailand; 7 Department of Clinical and Humanistic Psychology, Saybrook University, Pasadena, CA 91103 USA; 8 Department of Psychology, University of Missouri-Saint Louis, St. Louis, MO 63121 USA

**Keywords:** Psychology, Health care, Medical research

## Abstract

The experiences of close relationships-revised (ECR-R) is a widely used 36-item self-report measurement for measuring adult attachment. However, various short versions of the ECR-R have been developed and tested psychometrically. Given the cultural impact, a short version of the Thai ECR-R should be derived from the existing Thai version of the ECR-R. This study aimed to develop a 10-item version of the ECR-R that demonstrates comparable psychometric properties to the previous Thai version and the 18-item ECR-R. This study included four studies with a total of 1,322 participants. In study 1, 434 adults in a nonclinical setting were used for the development of the 10-item Thai ECR-R and tested in an independent sample. Studies 2, 3, and 4 were conducted on 312 adults in the clinical setting, 227 older adults in the nonclinical, and 123 older adults in clinical settings. The Cronbach alphas and corrected correlations between the ECR-R-18 and the ECR-R-10 in each study were calculated. Confirmatory factor analysis of the first-order two-factor solution model with fit statistics was examined with each sample. Correlations of the ECR-R-18 and the ECR-R-10 with other measurements were presented and compared. Known-group validity and measurement invariance test were also examined. The Cronbach alphas of the ECR-R-10 among all samples were acceptable, ranging between .77 and .85 for avoidance subscales and between .82 and .86 for anxiety subscales. The corrected correlation between the ECR-R-18 and ECR-R-10 was between .61 (*p* < .001) and .82 (*p* < .001). The values of the comparative fit index and Tucker-Lewis index for the model of ECR-R-10 were between .903 and .985, whereas the root-mean-square error of approximation was between .082 and .036, indicating that the model fits were acceptable. The ECR-R-10 was related to the measurements with a similar construct; however, no difference in the magnitude of correlation was observed between ECR-R-18 and ECR-R-10. Known group validity was established. Measurement invariance was successfully established across different age and gender groups, although it was only partially achieved with respect to clinical status. The ECR-R-10 provided equal or superior psychometric properties to the ECR-R-18 across age groups and settings. As it is a briefer scale, the ECR-R-10 can be practically used in general and clinical samples to reduce the burden of assessment, especially with older adults. Further investigation is needed to test the scale's temporal stability.

## Introduction

Over the past three decades, the adult attachment field has grown remarkably, becoming a pivotal focus in psychology and branching into diverse disciplines, notably medicine. The reason for this increasing emphasis on attachment is that it has been found to impact individuals at multiple levels, such as personality, coping styles, relationships, and health. Insecure attachment has been identified as a predisposing factor for a range of mental, physical, and interpersonal difficulties, including anxiety, depression, personality disorders, chronic diseases, relationship issues, substance abuse, and internet addiction. Attachment-related concerns also affect the working alliance in psychotherapy and may serve as a predictive factor for the success of therapy. Alongside clinician-administered measures, numerous self-report questionnaires have been developed to assess attachment in practical settings. A recent review has identified over 20 self-report questionnaires that have been utilized for this purpose^1^.

The Experiences in Close Relationships (ECR), a common self-report measurement used to assess attachment in adults, was developed by Brennan, Clark, and Shaver^2^. The original 323-item dataset was re-analyzed by Fraley and colleagues using item response theory to become a revised version with 36 items^3^.

It is widely recognized that the use of questionnaires represents a valuable method for assessing the construct of attachment. Specifically, the anxiety and avoidance subscales have demonstrated a high level of validity in measuring this construct. Attachment-related anxiety refers to an individual's fear of being rejected by others, which is associated with a need for excessive attention and a tendency to exhibit intense stress reactions when a partner is unavailable or indifferent. On the other hand, attachment-related avoidance involves a tendency to reject dependency and interpersonal proximity, accompanied by self-absorption and a reluctance to disclose personal information. Individuals who receive high scores on one or both of these subscales are typically considered to have an insecure attachment style, while those who score low on both anxiety and avoidance subscales are generally regarded as having a secure attachment style^4^. The ECR-R has been used and shown to be a significant predictor of mental health and psychiatric problems such as stress, anxiety, and social avoidance^5–7^.

The ECR-R (Experiences in Close Relationships-Revised) was originally developed and tested for its psychometric properties using Anglo-Saxon populations^2,3^. It has been tested and found to be acceptable with many other languages such as Greece, Hungarian, and Italian, including Thai samples^8–11^. Since the ECR-R has 36 items, shorter versions of ECR-R were developed. Based on different cultural backgrounds^12^, the valid attachment items for each developed measurement are different, despite the fact that they designed to elicit the same meaningful construct (i.e., anxiety and avoidance). The shorter version of the ECR-R included a 12-item scale (English)^13^, a 18-item Thai version, 12-item German version^14^, a 16-item Czech version^15^, and 14-item Slovak version^16^. The items of the short Thai ECR-R is different from the 12-item questionnaire used in Wei’s study^13^, and from a 12-item version of German ECR-R^14^. For example, only two items are the same between the Thai and German versions: "I often worry that my partner will not want to stay with me." and "I worry that romantic partners won't care about me as much as I care about them." Both are from the anxiety subscale, whereas none of the items is the same between the Thai and German versions for the avoidance subscale. This discrepancy may underscore cultural differences that influence the validity of them. All shorter versions, even in different languages, have demonstrated an acceptable fit for the model.

The 18-item Thai ECR-R (ECR-R-18) has been used in both clinical and nonclinical samples, with ages ranging from 18 to 95 years. The ECR-R-18 has been employed in several research studies to predict psychological problems such as loneliness, anxiety, depression, and suicidality^17–25^. Model misfit mainly resulted from one negative item (i.e., “I prefer not to show a partner how I feel deep down”), an item in avoidance subscale. This may be due to cultural influences. As found in related studies, Thai (or other Asian) respondents are more prone than western people to have difficulty responding negative items^26,27^. Therefore, a revision of the ECR-R-18 is needed. In addition, a revised ECR-R should be shorter when administered to older people. In summary, the 18-item Thai ECR-R demonstrates certain psychometric issues and is excessively lengthy.

The primary objective of this study was to assess the psychometric properties of the revised Thai version of the Experiences in Close Relationships scale across diverse samples, including young adults, older adults, and individuals from both nonclinical and clinical settings. We aimed to investigate whether the newly developed Thai ECR-R would demonstrate comparable or improved validity and reliability when compared to the previous 18-item version of the Thai ECR-R. Based on our hypothesis, we anticipated that the new Thai ECR-R would exhibit equivalent or superior psychometric properties in terms of validity and reliability compared to its predecessor. By examining a range of samples and settings, we aimed to obtain a comprehensive understanding of the revised scale's performance and its potential suitability for assessing attachment-related experiences in the Thai population.

## Materials and methods

A secondary analysis was conducted on data from several projects that used the ECR-R-18 questionnaire, which is detailed elsewhere^21,28–30^. The study involved four samples from four studies comprising adults in both clinical and non-clinical settings, as well as older adults in both clinical and non-clinical settings.

### Determining the number of items in the new ECR-R

As per the recommended guidelines, it is advised to include a minimum of four items per scale (eight in total) to ensure the reliability of measurements^31^. In line with this, we have the flexibility to choose between 8, 10, or 12 items, maintaining an equal number across each subscale. Although all three options are viable, opting for twelve items would offer a more comprehensive and robust measure compared to 10 or 8. However, after careful consideration, we have decided to strike a balance between the extremes and settle on 10 items for our study. These can be done simply by selecting the highest loading items for each subscale (avoidance and anxiety). Choosing items based on their loading values has several benefits, (1) construct validity: Items with high loadings are more likely to accurately represent and measure the intended construct, enhancing the validity of the scale. (2) reliability: Including items with high loadings increases the internal consistency and reliability of the scale. These items tend to contribute the most to the overall measurement precision of the construct. (3) efficiency: focusing on items with high loadings allows for the selection of a smaller subset of items that capture the essence of the construct, making the scale more concise and efficient. In addition, a new measurement should also be less burdensome, especially for older people. The 10-item ECR-R consists of five items for the anxiety subscale, and five items for the avoidance subscale.

### Participants

Study 1: 434 adults in a nonclinical sample was used to determine which 10 items should be used for the short version (ECR-R-10) using exploratory factor analysis. The rationale behind using a nonclinical sample for our study is rooted in its proximity to the general population. The original development of the ECR-R scale was based on data collected from a general population rather than clinical populations, although it has been found applicable to clinical samples as well. The five items with the highest factor loadings of each subscale were selected to compose the ECR-R-10. Then 226 university students were used to examine the construct validity of the ECR-R-10 using confirmatory factor analysis.

- Study 2: 312 The ECR-R-10 was examined in adults in clinical settings.
- Study 3: The ECR-R-10 was examined in 227 non-clinical older adult sample.
- Study 4: The ECR-R-10 was examined in 123 clinical older adult sample.

### Measurements

The following measurements were used along with the ECR-R in each study.

Study 1, *Rosenberg Self-Esteem Scale (RSES)*, a 4-point Likert scale. The Thai RSES revealed good internal consistency (α = 0.87). *State -Trait Anxiety Inventory (STAI) scale*, a trait -anxiety scale developed with a 20 -item instrument used to explore trait -anxiety. It is a 4-point scale. The Thai version of STAI used in this study showed good internal consistency (α = 0.89).

Study 2, *Perceived Stress Scale-10 (PSS-10)*, an instrument that measures the degree to which life events are perceived as being stressful. This is a 5-point Likert scale instrument. The Thai version of the PSS-10 has demonstrated good internal consistency (overall α = 0.85). *The Multi-dimensional Scale of Perceived Social Support (MSPSS)*, a 12-item, 7-Likert scale self-reporting tool that records how much social support respondents require. The Thai version has demonstrated an acceptable reliability (α = 0.90).

Study 3 *Revised UCLA Loneliness Scale*, a 6-item questionnaire (RULS-6) and uses a 4-point Likert scale. The Thai version has demonstrated an acceptable reliability (α = 0.75). *The Multi-dimensional Scale of Perceived Social Support (MSPSS)*, a 12-item, 7-Likert scale self-reporting tool which records how much social support respondents require. The Thai version has demonstrated an acceptable reliability (α = 0.92).

Finally, Study 4 used the *Perceived Stress Scale-10 (PSS-10)*, an instrument that measures the degree to which life events are perceived as being stressful. This is a 5-point Likert scale instrument. The Thai version of PSS-10 has demonstrated good internal consistency (overall α = 0.85). *The Multi-dimensional Scale of Perceived Social Support (MSPSS)*, a 12-item, 7-Likert scale self-reporting tool that records how much social support respondents require. The Thai version has demonstrated acceptable reliability (α = 0.90). We also used the *Core Symptoms Index (CSI)*, an instrument measuring anxiety, depression, and somatization. It consists of 15 items, all of which are based on a 5-point Likert scale. The CSI revealed a good internal consistency (α = 0.91).

### Statistical analyses

Descriptive statistics were used for data screening; all items revealed skewness and kurtosis <  ± 2)^32^. Missing values were managed by replacing them with the series’ mean. Exploratory factor analysis was carried out using a method of principal axis factoring for extraction, and varimax with Kaiser Normalization for rotation method. Confirmatory factor analysis was applied to determine a two-factor solution model, using the maximum likelihood method with oblique rotation. Model fit was evaluated using several indices, including the Comparative Fit Index (CFI) ≥ 0.95, the Tucker-Lewis Index (TLI) ≥ 0.9, the root-mean-square error of approximation (RMSEA) ≤ 0.6, and the standardized root-mean-square residual (SRMR) ≤ 0.08^33^.

To determine convergent and discriminant validity, Pearson's correlation coefficients were calculated between the ECR-R and other measures. To account for spurious correlation in the evaluation of the short-form, the corrected correlation coefficients obtained by Levy’s method were used to calculate the correlation coefficients between ECR-R-18 and ECR-R10^34^. Known group validity was determined by calculating the difference of ECR-R scores between nonclinical and clinical groups. Finally, internal consistency reliability was assessed by calculating the Cronbach's α coefficient, with a reliability of more than 0.70 considered acceptable. Test–retest reliability was performed using intraclass correlation in nonclinical sample. These analyses were conducted using IBM SPSS version 22 and AMOS package version 22.

To determine the invariance of the new ECR-R, we performed multigroup CFA (MGCFA) between sexes on each sample (adult and older adult), and among four groups using confirmatory factor analyses and measurement invariance analyses. Based on Byrne^35^, the following fit criteria of the factorial model were applied, CFI and TLI ≥ 0.90, SRMR < 0.05 and RMSEA ≤ 0.1^36^. In multigroup CFA, Goodness-of-fit statistics were estimated for each model and for each model relative to the previous, less restricted, model. The ΔCFI and ΔRMSEA between the more and less constrained models were evaluated. The ΔCFI and △TLI larger than 0.01 and ΔRMSEA larger than 0.015 indicated a significant worsening of fit^37^. This analysis was performed with Mplus 8.10 version.

### Ethics approval 

The study was conducted according to the guidelines of the Declaration of Helsinki and approved by the Institutional Review Board (or Ethics Committee) of the Faculty of Medicine, Chiang Mai University (EC: PSY-2566–0110, approved date 11 April 2023).

### Consent to Participate

Informed consent was obtained from all individual participants included in the study.

## Results

The respondents’ characteristics of each sample follow. Study 2: 276 adults in clinical setting (77.2% female, mean age 39.88 [SD 11.95]), all diagnosed with depressive disorder. Study 3, 236 non-clinical older adult sample (57.6% female, mean age 73.52 (SD 7.32), and Study 4, 803 clinical older adult sample (69.7% female, mean age 69.24 (SD 6.88), 23.7% depressive disorder).

Study 1. ECR-R-10 derived from 434 adults in non-clinical sample (58.1% female, mean age 18.9 [SD 1.15]). The 10 items drawn from the 18 items are shown in Table S1. Cronbach’s alpha of each subscale was 0.85 and 0.81 for avoidance and anxiety, respectively (Table 1). The corrected correlation between ECR-R-18 and ECR-R10 was 0.82 (*p* < 0.01) for avoidance and 0.75 (*p* < 0.01) for anxiety subscales (Table 2). Avoidance and anxiety subscales were significantly related to STAI (r = 0.12, *p* < 0.05, and r = 0.281, *p* < 0.01, respectively), whereas only the anxiety subscale was significantly related to RSES (r = 0.14, *p* < 0.01) (Table 3). No significant difference was observed between STAI or RSES or the subscales of avoidance and anxiety of the ECR-R-18, and ECR-R-10. Test -retest reliability assessed by the intra-class correlation coefficient (ICC) during the six -week retest period showed that the ICC value was 0.759 (95% CI 0.651,0.831) for avoidance subscale, and 0.790 (95% CI 0.705, 0.850) for the anxiety scale.

**Table 1:** Cronbach’s alpha of avoidance and anxiety subscales of ECR-R-18 and ECR-R-10.

	ECR-R-18		ECR-R-10	
	Avoidance	Anxiety	Avoidance	Anxiety
Study 1: adults, nonclinical	.90	.88	.85	.81
Study 2: adults, clinical	.79	.89	.81	.86
Study 3: older adults, nonclinical	.74	.90	.81	.83
Study 4: older adults, clinical	.74	.88	.77	.82

**Table 2:** Mean and standard deviation, Pearson’s and Levy’s correlation of the two versions of the ECR-R.

Study	ECR-R-18		ECR-R-10		ECR-R-18		ECR-R-10	
	Avoidance	Anxiety	Avoidance	Anxiety	Avoidance	Anxiety	Avoidance	Anxiety
	Mean ± SD	Mean ± SD	Mean ± SD	Mean ± SD	Pearson’s correlation	Levy’s Corrected correlation	Pearson’s correlation	Levy’s corrected correlation
Study 1: adults, nonclinical	2.93 ± 1.20	3.27 ± 1.20	2.94 ± 1.25	3.34 ± 1.29	0.972	0.816	0.951	0.747
Study 2: adults, clinical	3.86 ± 1.22	3.74 ± 1.51	3.91 ± 1.55	3.85 ± 1.67	0.949	0.708	0.951	0.759
Study 3: older adults, nonclinical	3.55 ± 1.04	3.40 ± 1.14	2.91 ± 1.18	3.78 ± 1.41	0.934	0.718	0.963	0.786
Study 4: older adults, clinical	3.74 ± 1.16	2.92 ± 1.00	3.74 ± 1.16	3.97 ± 1.00	0.868	0.612	0.969	0.772

SD = standard deviation.

**Table 3:** Correlation coefficients between avoidance and anxiety subscales of ECR-R-18 and ECR-R-10.

	Measurements	ECR-R-18		ECR-R-10	
		Avoidance	Anxiety	Avoidance	Anxiety
Study 1: adults, nonclinical	RSES	− .070	− .160**	− .056	− .140**
	STAI	.131**	.298**	.120*	.281**
Study 2: adults, clinical	PSS-10	.068	.341**	.084	.330**
	MSPSS	− .241**	− .126*	− .228**	− .130*
Study 3: older adults, nonclinical	RULS-6	.035	.199**	.031	.168**
	MSPSS	− .509**	.170**	− .474**	.159**
	GDS	.036	.322**	.025	.297**
Study 4: older adults, clinical	PSS-10	− .025	.183	− .042	.160
	MSPSS	− .019	− .045	− .065	− .033
	CSI	.069	.314**	.169	.283*

RSES = Rosenberg self-esteem scale, STAI = State trait anxiety inventory, PSS-10 = Perceived stress scale, MSPSS = Multidimensional scale of perceived social support, RULS-6 = Revised UCLA loneliness scale, GDS = Geriatric depression scale, CSI = Core symptom index.

**Figure 1:**
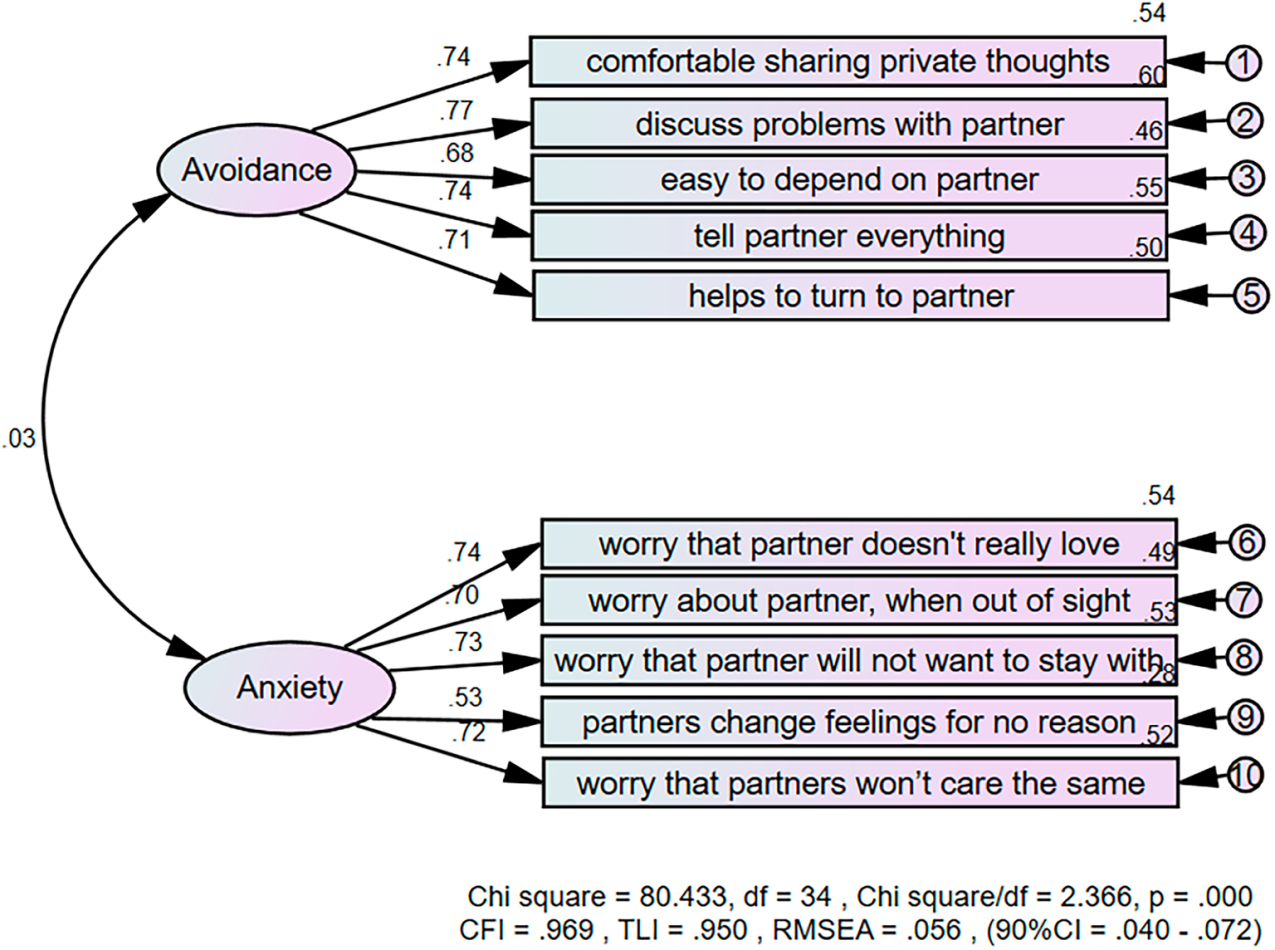
The first order two-factor solution model of the 10-item Thai version of ECR-R with factor loadings and fit indices. CFI = comparative fit index, TLI = Tucker-Lewis Index, RMSEA = root-mean-square error of approximation, SRMR = standardized root-mean-square residual.

CFA results yielded factor loadings between 0.68 and 0.74 for the avoidance construct, 0.53 and 0.74 for the anxiety construct. A two-factor solution with the fit indices as follows, CFI = 0.969, TLI = 0.950, RMSEA = 0.056 (90% CI = 0.40, 0.072), suggested that the two-factor solution model had an excellent fit to the data (Fig. 1) (Table 5).

**Table 4:** Mean and standard deviation of the two versions of the ECR-R between Nonclinical and clinical groups (n = 1008).

Study	ECR-R-18		ECR-R-10	
	Avoidance	Anxiety	Avoidance	Anxiety
	Mean ± SD	Mean ± SD	Mean ± SD	Mean ± SD
Nonclinical (n = 604)	3.91 ± 1.10	3.46 ± 1.16	2.88 ± 1.16	3.44 ± 1.28
Clinical (n = 404)	3.07 ± 1.12	3.99 ± 1.42	3.69 ± 1.55	4.00 ± 1.60
Test difference	*t* (1006) = 11.724,* p* < .001	*t* (699) = 6.145,* p* < .001	*t* (699) = 8.987,* p* < .001	*t* (732) = 5.839,* p* < .001

SD = standard deviation.

To ensure its construct validity, the ECR-R-10 was explored in a new sample of 224 university students (52.9% female, mean age of 20.43 [SD = 0.65]), and the CFA results yielded factor loadings between 0.48 and 0.83 for the avoidance construct, and 0.59 and 0.78 for the anxiety construct. A two-factor solution with the fit indices as follows, CFI = 0.955, TLI = 0.939, RMSEA = 0.068 (90% CI = 0.45, 0.092), and SRMR = 0.0677, suggesting that the two-factor solution model was a good fit for the data.

Study 2. ECR-R-10 was examined in 312 adults in a clinical sample (77.2% female, mean age 39.88 [SD 11.95]), all diagnosed depressive disorder. Cronbach’s alpha of each subscale was 0.85 and 0.86 for avoidance and anxiety, respectively (Table 1). The corrected correlation between ECR-R-18 and ECR-R10 was 0.708 (*p* < 0.01) for avoidance and 0.759 (*p* < 0.01) for anxiety subscales (Table 2). Avoidance and anxiety subscales were significantly related to MSPSS scores (r = − 0.228, *p* < 0.01, and r = − 0.130, *p* < 0.05, respectively), whereas only anxiety subscale was significantly related to PSS scores (r = 0.330, *p* < 0.01) (Table 3). No significant difference was observed between the relationship between MSPSS or PSS and the subscales of avoidance and anxiety of the ECR-R-18, and ECR-R-10.

CFA results yielded factor loadings between 0.49 and 0.88 for avoidance construct, 0.59 and 0.84 for anxiety construct. A two-factor solution with the fit indices as follows, CFI = 0.955, TLI = 0.925, RMSEA = 0.082 (90% CI = 0.062, 0.102), suggesting that the two-factor solution model had a good fit to the data (Table 5).

Study 3. ECR-R-10 was examined in 236 non-clinical older adult sample (57.6% female, mean age 73.52 [SD7.32]). Cronbach’s alpha for each subscale was 0.81 and 0.83 for avoidance and anxiety, respectively (Table 1). The corrected correlation between ECR-R-18 and ECR-R10 was 0.718 (*p* < 0.01) for avoidance and 0.786 (*p* < 0.01) for anxiety subscales (Table 2). Avoidance and anxiety subscales were significantly related to MSPSS scores (r = − 0.474, *p* < 0.01, and r = − 0.159, *p* < 0.01, respectively), whereas only anxiety subscale was significantly related to RULS scores (r = 0.159, *p* < 0.01) (Table 3). No significant difference was observed between the relationship between MSPSS or RULS and the subscales of avoidance and anxiety of the ECR-R-18, and ECR-R-10.

CFA results yielded factor loadings between 0.61 and 0.76 for avoidance construct, 0.59 and 0.84 for anxiety construct. A two-factor solution with the fit indices as follows, CFI = 0.953, TLI = 0.925, RMSEA = 0.077 (90% CI = 0.053, 0.101), suggested that the two-factor solution model had a good fit to the data (Table 5).

Study 4. ECR-R-10 was examined in 803 older adults in clinical settings (69.7% female, mean age 69.24 [SD 6.88], 23.7% depressive disorder). Cronbach’s alpha of each subscale was 0.77 and 0.82 for avoidance and anxiety, respectively (Table 1). The corrected correlation between ECR-R-18 and ECR-R10 was 0.612 (*p* < 0.01) for avoidance and 0.772 (*p* < 0.01) for anxiety subscales (Table 2). Anxiety subscale was significantly related to CSI total scores (r = 0.283, *p* < 0.05) (Table 3). No significant difference was observed between the relationship between MSPSS or PSS and the subscales of avoidance and anxiety of the ECR-R-18, and ECR-R-10.

CFA results yielded factor loadings between 0.44 and 0.87 for avoidance construct, 0.63 and 0.87 for anxiety construct. A two-factor solution with the fit indices as follows, CFI = 0.985, TLI = 0.980, RMSEA = 0.036 (90% CI = 0.000, 0.092), suggesting that the two-factor solution model was an excellent fit to the data (Table 5).

Table 1 shows that no significant difference of Cronbach’s alpha values between ECR-R-18 and ECR-R-10 in each sample.

Table 2 shows that no significant difference between ECR-R-18 and ECR-R-10 in each sample. The correlation between the ECR-R-18 and ECR-R-10 ranged from 0.868 to 0.972. However, when bias was corrected the ranges were between 0.612- 0.816.

Table 3 shows the correlation coefficients between other measurements and ECR-R-18 and ECR-R-10. No difference between all of the correlation coefficients between avoidance and anxiety subscales of ECR-R-18 and ECR-R-10 were observed. For example, the z-statistics of the difference of correlation coefficients between RSES and the anxiety subscales of ECR-R-18 and ECR-R-10 was 0.288, *p* = 0.773 (95% CI − 0.118, 0.158).

Table 4 shows the significant difference of ECR-R-18 and ECR-R-10 between nonclinical and clinical groups (*p* < 0.001), indicating known-group validity.

Table 5 shows the results of the first order two-factor solution model proposed for the samples. It was found that the model fit was better in nonclinical samples than clinical samples, and better in adults than older participants. The models from the 10-ECR-R yielded better fit indices than from the 18-ECR-R. Item 1 (“I prefer not to show a partner how I feel deep down”) appeared to be a problem for Thai participants, especially older participants.

**Table 5:** Factor loadings of avoidance and anxiety subscales of the ECR-R-10 and the fit indices of the first order two-factor solution model.

Study	Version	Factor Loading		Fit index			
		Avoidance	Anxiety	Chi-square/df	CFI	TLI	RMSEA (90%CI)
Study 1: adults, nonclinical	ECR-R-18	0.64–0.76	0.55–0.78	409.86/134	.921	.899	.069(.061, .072)
	ECR-R-10	0.68–0.74	0.53–0.74	80.433/34	.969	.950	.056(.040, .072)
Study 2: adults, clinical	ECR-R-18	0.14–0.78	0.50–0.77	336.191/118	902	.873	.077(.067,.087)
	ECR-R-10	0.49–0.88	0.63–0.78	83.612/27	.955	.925	.082(.062.102)
Study 3: older adults, nonclinical	ECR-R-18	0.46–0.77*	0.65–0.85	236.518/115	.928	.905	0.068(.056, .081)
	ECR-R-10	0.61–0.76	0.59–0.84	65.435/28	.953	.925	.077(0.053, 0.101)
Study 4: older adults, clinical	ECR-R-18	0.17–0.76	0.52–0.83	178.295/123	.922	.903	.061(.040,.080)
	ECR-R-10	0.44–0.87	0.63–0.87	38.251/33	.985	.980	.036(.000,.092)

*− .35 for item1, CFI = comparative fit index, TLI = Tucker-Lewis index, RMSEA = the root-mean-square error of approximation, CI = confidence interval.

Test–retest reliability was performed using intraclass correlation in study 1. For the ECR-R-18, the intraclass coefficient was 0.70 (*p* < 0.0001) for anxiety, 0.50 (*p* < 0.0001) for avoidance. For the ECR-R-10, the intraclass coefficient was 0.79 (*p* < 0.0001) for anxiety, 0.76 (*p* < 0.0001) for avoidance.

Table 6 presents the fit measures of the multi-group models used to test measurement invariance across different age groups, sexes, and clinical statuses. The assumption was that measurement invariance held when the fitting change met the following conditions: nonsignificant Chi-square test, △CFI ≤ 0.01, △TLI ≤ 0.01, and △RMSEA < 0.015. The results indicated that measurement invariance was suggested across sexes and age groups (adult vs. older adult). However, in terms of clinical status, the data supported both the configural invariance and metric invariance models, but not the scalar invariance model. This finding remained consistent when comparing all four groups: adult nonclinical, adult clinical, older adults nonclinical, and older adult clinical groups (Δχ2 (Δdf) = 123.553(24), *p* < 0.001, ΔCFI = 0.022, ΔTLI = 0.018, ΔRMSEA = 0.013).

**Table 6:** Model comparisons for measurement invariance testing across sex, age, and clinical status.

Model	χ^2^(*df*)	Δχ^2^(Δ*df*)	CFI	TLI	SRMR	RMSEA	ΔCFI	ΔTLI	ΔRMSEA
Invariance across sex groups									
Model 1	317.514 (68)		0.933	0.911	0.051	0.085			
Model 2	328.447(76)	10.933(8),* p* = 0.205	0.932	0.919	0.054	0.081	0.001	0.008	0.004
Model 3	338.969(84)	10.521(8),* p* = .1617	0.931	0.926	0.038	0.055	0.001	0.007	0.026
Invariance across age groups									
Model 1	148.424 (56)		0.948	0.916	0.076	0.090			
Model 2	150.739(64)	2.314(8),* p* = 0.9698	0.951	0.931	0.079	0.081	0.003	0.015	0.009
Model 3	161.902(72)	10.521(8),* p* = .1926	0.949	0.936	0.084	0.078	0.002	0.005	0.003
Invariance across clinical status groups									
Model 1	184.282(56)		0.971	.953	.048	.067			
Model 2	196.297(64)	12.015(8),* p* = 0.15	0.970	.958	.053	.064	0.001	0.003	0.003
Model 3	260.461(72)	64.163(8),* p* < .001	0.957	.947	.061	.071	0.013	0.011	0.007
Invariance across four groups (adult nonclinical, adult clinical, older adults nonclinical, and older adult clinical samples)									
Model 1	247.066(112)		0.970	.952	.054	.069			
Model 2	274.431(136)	27.365(24),* p* = 0.2877	0.969	.959	.062	.063	0.001	0.007	0.006
Model 3	397.984(160)	123.553(24),* p* < .001	0.947	.941	.073	.076	0.022	0.018	0.013

Model 1, configural invariance; Model 2, metric invariance; Model 3, scalar invariance; Model 4, residual invariance. χ2, chi-squared test; df, degrees of freedom; CFI, comparative fit index; TLI, Tucker-Lewis index; SRMR, standardized root mean square residual; RMSEA, root mean square error of approximation.

## Discussion

In general, the abbreviated Thai version of the ECR-R-10 demonstrates a significant correlation with the ECR-R-18 but offers superior psychometric properties. This can be attributed to the removal of a bad fit item, specifically item 1 (i.e., "I prefer not to show a partner how I feel deep down"). The 10-item version has been shown to possess construct validity within four distinct populations, including nonclinical and clinical adults, as well as nonclinical and clinical older adults. However, it is noteworthy that the model fit among older adults appears to be comparatively lower than in younger adults. In summary, the abbreviated Thai version of the ECR-R-10 has exhibited satisfactory reliability and validity for utilization in both age groups and various settings. Regarding construct validity, the data align well with a two-factor solution model, and concurrent validity as well as known-group validity have been supported. Furthermore, in addition to the observed acceptable to excellent levels of internal consistency as indicated by Cronbach's alphas, the nonclinical sample of the initial study demonstrated favorable results for test–retest reliability over a six-week timeframe.

When compared to other short versions of the ECR-R in English, Czech, Spanish, German, and Korean^38^, it is expected that the items of the ECR-R are different. Three out of five of the avoidance items and two out of five of the anxiety items are consistent with the Korean version, whereas four out of five (80%) of the Thai ECR-R-10 items are included in the 18-item Spanish version. This may be attributable to cultural differences in how respondents perceive avoidance and anxiety in relationships, as well as the characteristics of the sample. In terms of internal consistency, all shorter versions developed including the present study are good to excellent. Like the ECR-RD8 (tested in German-speaking countries: Germany, Austria, and Switzerland), the first order two-factor solution model in the current study fitted the data the best.

Unlike other studies that were mostly conducted using nonclinical samples, the present study was conducted in both clinical and nonclinical settings using the same set of items. Notably, item 1 (i.e., "I prefer not to show a partner how I feel deep down"), which was included in the 18-item Thai version, seems to have a poor fit to the model and is not included in other versions. The fact that this item does not sufficiently contribute to the construct when compared to other items makes it difficult to draw definitive conclusions. One possible hypothesis is that this variance results from the fact that this item is the only item that has different direction from the rest. In addition, it is the only item that is negatively worded^27,39^.

The ECR-R-10 demonstrated measure invariance across sexes and age but not in different clinical status. This suggests that there are differences in the scaling or measurement units between the two groups, but the underlying structure or relationships between the observed variables and latent constructs are similar. The failure of scalar invariance implies that the measurement scales are not comparable between the nonclinical and clinical samples. The observed scores in the two groups might be influenced by factors specific to each group, such as differences in symptom severity, response biases, or other unexplored contextual factors related to the clinical condition.

### Clinical implications

The findings of this study indicate that the ECR-R-10, in both nonclinical and clinical populations, demonstrated strong validity and reliability. Therefore, this shorter version of the questionnaire can be effectively utilized in a wide range of applications. Notably, the ECR-R-10 exhibited superior construct validity when compared to the longer ECR-R-18 version. Given its robust psychometric properties, the 10-item version is a viable and recommended alternative to the 18-item questionnaire. Researchers and practitioners can confidently use the ECR-R-10 as a reliable measure of attachment-related constructs while enjoying the benefits of its brevity and efficient administration.

### Limitations and future research

Despite the merits of brevity and wide use, the ECR-R-10 has some limitations. First, the sample used for analysis might not be representative of nonclinical adults because most participants were young adults, whereas the clinical sample used in this study was confined to psychiatric outpatient, mostly those with depressive disorders. It cannot represent all ranges of clinical samples. Secondly, it is important to note that the term 'clinical' used in this study was quite broad and encompassed participants with various diagnoses and levels of severity. This broad categorization might pose challenges when conducting measurement invariance testing. Therefore, it is recommended that future research endeavors employ a more specific and refined classification for clinical status and explore the possibility of developing group-specific measurement models. Thirdly, test–retest reliability has not been fully examined in all studies, and further investigation of this stability should be conducted in other populations, particularly in clinical settings. Lastly, it is worth noting that the present study utilized multiple instruments in addition to the ECR-R questionnaire. This inclusion of diverse instruments to validate the questionnaire across different populations and time frames may have influenced the study's results. Therefore, it is imperative to conduct replication studies to comprehensively examine the set of items used in the questionnaire. By replicating the study and utilizing consistent instruments, we can further validate the findings and ensure the robustness of the questionnaire's items.

## Conclusion

The ECR-R-10 provided sufficient psychometric properties across nonclinical and clinical adult and older adult cohorts. As it is brief, it can be administered to many age groups with less burden, especially older adults. Testing for the scale's stability, such as test–retest reliability, should be further investigated.

### Supplementary Information

Supplementary Information.

## Data Availability

The datasets used and/or analyzed during the current study are available from the corresponding author on reasonable request.
