# Supplementary material for: Psychometric assessment of the 10-item, revised experience of close relationship (ECR-R-10) in nonclinical and clinical populations of adults and older adults in Thailand
Source: Sci Rep. 2023 Sep 11;13:14969. doi: 10.1038/s41598-023-41306-y (PMC10495348; doi:10.1038/s41598-023-41306-y)
Supplement: Supplementary file 1 — Supplementary Information. [file 41598_2023_41306_MOESM1_ESM.docx]

| Table S1. 10-item of the ECR-R-10 selected from ECR-R-18 |
| --- |
| *Anxiety* |
| **02. I often worry that my partner doesn't really love me.** |
| **04 When my partner is out of sight, I worry that he or she might become interested in someone else.** |
| 06. My romantic partner makes me doubt myself. |
| **08. I often worry that my partner will not want to stay with me.** |
| 10. I worry a lot about my relationships. |
| 12. When I show my feelings for romantic partners, I'm afraid they will not feel the same about me |
| **14. Sometimes romantic partners change their feelings about me for no apparent reason.** |
| **16. I worry that romantic partners won’t care about me as much as I care about them.** |
| 18 I worry that I won't measure up to other people. |
| *Avoidance* |
| 01. I prefer not to show a partner how I feel deep down. |
| **03. I feel comfortable sharing my private thoughts and feelings with my partner.** |
| 05. I find it relatively easy to get close to my partner. |
| **07. I usually discuss my problems and concerns with my partner.** |
| **09. I find it easy to depend on romantic partners.** |
| **11. I tell my partner just about everything.** |
| 13. It's not difficult for me to get close to my partner. |
| 15. I talk things over with my partner. |
| **17. It helps to turn to my romantic partner in times of need.** |
| Note: Bold = selected item |
